# Phenylalanine-Derived β-Lactam TRPM8 Modulators. Configuration Effect on the Antagonist Activity

**DOI:** 10.3390/ijms22052370

**Published:** 2021-02-27

**Authors:** María Ángeles Bonache, Pedro Juan Llabrés, Cristina Martín-Escura, Roberto De la Torre-Martínez, Alicia Medina-Peris, Laura Butrón, Isabel Gómez-Monterrey, Ana María Roa, Gregorio Fernández-Ballester, Antonio Ferrer-Montiel, Asia Fernández-Carvajal, Rosario González-Muñiz

**Affiliations:** 1 Instituto de Química Médica (IQM-CSIC), Juan de la Cierva 3, 28006 Madrid, Spain; angelesbonache@iqm.csic.es (M.Á.B.); pedrojuan637@gmail.com (P.J.L.); cristinamartinescura@gmail.com (C.M.-E.); 2 Alodia Farmacéutica SL, Santiago Grisolia 2, Tres Cantos, 28760 Madrid, Spain; ana.maria.roa.arranz@gmail.com; 3 IDiBE, Universidad Miguel Hernández, Avda. de la Universidad s/n, 03202 Elche, Spain; rober.dltm@gmail.com (R.D.l.T.-M.); mepea8@gmail.com (A.M.-P.); lbutron@umh.es (L.B.); gregorio@umh.es (G.F.-B.); aferrer@umh.es (A.F.-M.); asia.fernandez@umh.es (A.F.-C.); 4 Department of Pharmacy, University Federico II of Naples, 80131 Naples, Italy; imgomez@unina.it

**Keywords:** TRPM8, antagonists, β–lactams, absolute configuration, Ca^2+^ microfluorimetry, Patch-Clamp

## Abstract

Transient receptor potential cation channel subfamily M member 8 (TRPM8) is a Ca^2+^ non-selective ion channel implicated in a variety of pathological conditions, including cancer, inflammatory and neuropathic pain. In previous works we identified a family of chiral, highly hydrophobic β–lactam derivatives, and began to intuit a possible effect of the stereogenic centers on the antagonist activity. To investigate the influence of configuration on the TRPM8 antagonist properties, here we prepare and characterize four possible diastereoisomeric derivatives of 4-benzyl-1-[(3′-phenyl-2′-dibenzylamino)prop-1′-yl]-4-benzyloxycarbonyl-3-methyl-2-oxoazetidine. In microfluorography assays, all isomers were able to reduce the menthol-induced cell Ca^2+^ entry to larger or lesser extent. Potency follows the order 3*R*,4*R*,2′*R* > 3*S*,4*S*,2′*R ≅* 3*R*,4*R*,2′*S >* 3*S*,4*S*,2′*S*, with the most potent diastereoisomer showing a half inhibitory concentration (IC_50_) in the low nanomolar range, confirmed by Patch-Clamp electrophysiology experiments. All four compounds display high receptor selectivity against other members of the TRP family. Furthermore, in primary cultures of rat dorsal root ganglion (DRG) neurons, the most potent diastereoisomers do not produce any alteration in neuronal excitability, indicating their high specificity for TRPM8 channels. Docking studies positioned these β-lactams at different subsites by the pore zone, suggesting a different mechanism than the known *N*-(3-aminopropyl)-2-[(3-methylphenyl)methoxy]-*N*-(2-thienylmethyl)-benzamide (AMTB) antagonist.

## 1. Introduction

Transient receptor potential cation channel subfamily M member 8 (TRPM8), is a calcium-permeable ion channel, activated by innocuous cooling to cold temperatures (below 28 °C), membrane depolarization, changes in osmolarity and pH [1,2]. In addition, it is activated by chemical agents such as (−)-menthol and icilin, and by different endogenous molecules, such as the lipid PIP2 [3], testosterone [4], artemin [5] and Pirt (phosphoinositide interacting regulator of TRPs) [6]. TRPM8 is a cold-sensing multimodal nociceptor of the somatosensory nervous system and a major sensor of cold nociception in humans [7,8]. On one hand, they are highly expressed in primary sensory neurons (Aδ and C-fibers) of the dorsal root ganglia (DRG) and trigeminal ganglia (TG) [9], which participate in the sensory encoding of pain under both normal and pathological conditions. Accordingly, TRPM8 channels have been implicated in inflammatory pain, but also in migraine [10,11,12]. On the other hand, the cold allodynia associated to oxaliplatin-induced painful neuropathy has been correlated with TRPM8 expression and function [13,14], while other studies indicate that TRPM8 is implicated in cold allodynia after inflammation or nerve injury [15].

TRPM8 channels are also expressed on deep visceral afferents in bronchopulmonary tissue [16], bladder [17], prostate [18], brain [19], and other tissues such as in eyes [20], salivary glands [21] or the oropharyngeal system [22]. Recent experimental evidence seems to indicate that TRPM8 and its modulation by testosterone is behind the regulation of dimorphic sexual and social behaviors in mice [23]. Several tumor growth progression and invasion capacity initial phases of different cancers (prostate, pancreas, colon, breast, lung, and skin), have also been connected with aberrant expression of TRPM8 channels [24], and suggest a potential protective role for TRPM8 antagonists. Contrastingly, for advanced metastatic tumors, it seems that TRPM8 activation by agonists could have a protective function [25].

Among described TRPM8 agonists, we mainly found tertiary amides and diverse menthol derivatives [26,27,28], and some other natural products [29]. The family of TRPM8 antagonists is highly diverse, with either different acyclic (amide, sulfonamide, urea, glycine, tryptophan) or heterocyclic (tiazole, 2-azetidinone, benzothiophene, benzimidazole, isoquinoline) central scaffolds [27,28,30,31,32,33,34]. Despite the big number of TRPM8 modulators described to date, only a few have been extended to clinical studies, with the exception of menthol that has been profusely scrutinized as a topical antihyperalgesic agent. Among TRPM8 antagonists, only PF-05105679 and AMG 333 progressed into phase I clinical trials to explore pharmacokinetics safety and tolerability pharmacodynamics in healthy volunteers and migraneous patients, respectively [35,36]. Both compounds evidenced several adverse effects, including feeling hot, that preclude further progress into the clinic. Therefore, it is of great interest to push in the discovery of new, potent and selective TRPM8 antagonists, and to increase the knowledge about their specific binding sites on the target protein, to search for mechanistically different antagonists [37,38,39].

**Figure 1:**
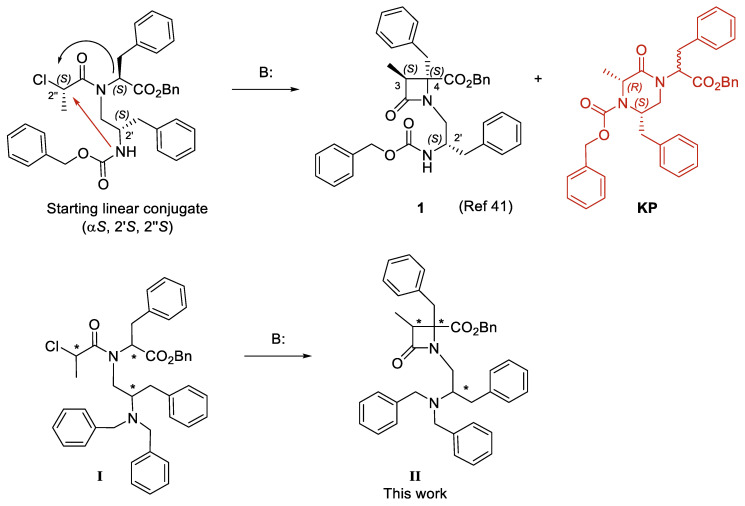
Previously described β–lactam (**1**) with transient receptor potential cation channel subfamily M member 8 (TRPM8) antagonist properties and analogues in this work (**II**), obtained from linear conjugates **I**. The black arrow denotes cyclization to β–lactam and red arrow to ketopiperazine (**KP**). * Means the position of stereogenic centers.

In a previous study, we described a library of highly substituted azetidin-2-ones, prepared from Phe and Asp/Glu amino acid conjugates, that potently and selectively block the activation of TRPM8 by voltage, menthol, and temperature [40]. The prototype β-lactams bear four hydrophobic substituents very important for activity, three benzyl groups and a *tert*-butoxycarbonyl (Boc) moiety, and a short *N*-alkyl chain (1–2 carbons). Very recently, we reported on the cyclization of a series of shorter phenylalaninol-Phe conjugated, affording 2-azetidinones and/or 2-ketopiperazine (KP) derivatives, depending on the *N*-chloroalkyl group and on the configuration of linear intermediates [41]. Cyclization of all *S*-configured linear choroppropanoyl derivatives afforded the expected β-lactam **1** along with a small quantity of its regiomeric 2-ketopiperazine (KP), due to the cyclization through the 2′-NH group (indicated by a red arrow, Figure 1). Depending on the configuration of the starting chloropropanoyls, the 2-KP species can be the predominant cyclization product. In addition, for 2″-*R*-chloropropanoyl derivatives, the corresponding β-lactams were obtained as inseparable mixtures of diastereoisomers, thus precluding the study on the configuration influence. The prototype β–lactam (**1**, configuration 3*S*,4*S*,2′*S*) blocks the channel activation by menthol (Ca^2+^ entry assay) with micromolar values, significantly reduces the oxaliplatin-induced allodynia from 15 to up to 60 min in a mice model in vivo, and exhibits non-selective antitumor activity (micromolar potency in four tumor cell lines) [41]. More importantly, docking studies indicated that compound **1** localizes preferentially at two possible locations by the pore zone of the TRPM8 channel, suggesting either an allosteric or a pore blocking mechanism, different to that of other described antagonists.

To avoid the regioisomeric formation of diastereomeric KPs, and thus making the synthetic approach to this type of β-lactams more efficient, here we propose the substitution of the Z-group in **1** by an NBn_2_ moiety (general formula **II**, Figure 1), lacking the reactive 2′-NH group in linear precursors **I**. The dibenzylamino moiety is present in other potent tryptamine- and tryptophan-derived TRPM8 antagonists [32,33], and some conformationally restricted tetrahydro-β-carboline derivatives [42]. The 3*S*,4*S*,2′*R*-diastereoisomer of β–lactam **1** showed slightly better TRPM8 antagonist activity than the *N*-2’S-isomer (**1**), while the corresponding 3*R*,4*R*,2′*S*- and 3*R*,4*R*,2′*R*-isomers could not be obtained in diasteroisomeric pure form [41]. Given the intrinsic chirality of biological systems, the absolute configuration of drugs is normally an important issue. Therefore, to assess the influence of the configuration on the TRPM8 antagonist activity in this family of compounds, we first prepare enantiopure linear conjugates **I**, which were cyclized to four diastereoisomeric β-lactams of general structure **II**, those which are synthetically accessible in optical pure form linear precursors **I**. Then, these diastereomeric compounds were assayed for their potency as TRPM8 antagonists in a Ca^2+^ entry assay, the best antagonists were validated by Patch-Clamp experiments, and their selectivity over other TRPs was assessed. Finally, we have identified possible binding sites for these compounds in the TRPM8 channel through docking studies.

## 2. Results and Discussion

### 2.1. Chemistry

**Scheme 1:**
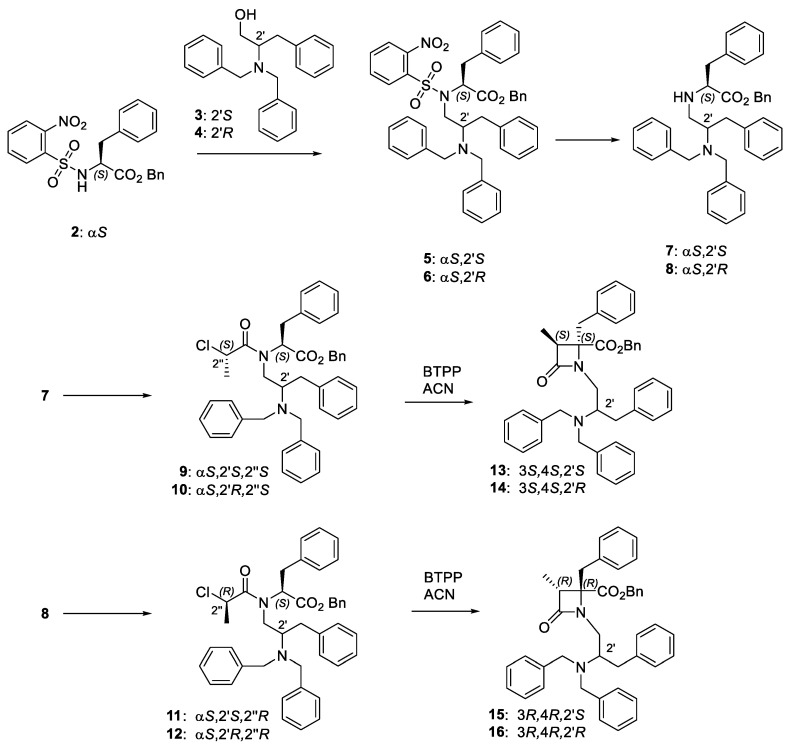
Synthetic pathway for the preparation of the four diastereoisomeric β-lactam derivatives.

*N*-Dibenzylphenylalaninol 2-azetidinones **13**–**16** were prepared from commercial alcohols **3** and **4** in three steps (Scheme 1), following the previously described synthetic procedure [41].

The synthesis of enantiopure 2-chloropropanoyl derivatives **9**, **10**, **11** and **12** was started by a Mitsunobu reaction between Ns-L-Phe-OBn (**2**) and the corresponding commercial alcohol **3** and **4** to afford compounds **5** and **6** (Scheme 1). Then, the removal of the nosyl group gave NH derivatives **7** and **8**. Finally, compounds **7** and **8** were reacted with 2S- or 2R-chloropropionic acid in the presence of trichloroacetonitrile and triphenylphosphine. We have previously reported that in the cyclization of enantiopure 2-chloropropanoyl derivatives to β-lactams, the stereochemistry at the C3,C4 bond is exclusively governed by the configuration of the chloropropanoyl moiety, with 2″*S*- and 2″-R-chloropropionyl derivatives affording 3*S*,4*S* and 3*R*,4*R* β–lactams, respectively [43]. These configurational results were rationalized through quantum mechanics calculation, which indicated that the energy of the possible transition states leading to ring-closure is highly favorable for the formation of *cis*-3-methyl-β-lactams, with >10 KJ/mol lower energy compared to the *trans*-diastereoisomers [44]. Therefore, the phosphazene base P_1_-*t*-Bu-tris(tetramethylene) (BTPP)-assisted cyclization of linear 2″*S*-conjugates **9** (α*S*,2′*S*,2″*S*) and **10** (α*S*,2′*R*,2″*S*) afforded *cis*-3-methyl 3*S*,4*S*-β-lactams **13** (3*S*,4*S*,2′*S*) and **14** (3*S*,4*S*,2′*R*), respectively. In a similar manner, *cis*-3-methyl 3*R*,4*R*-β-lactams **15** (3*R*,4*R*,2′*S*) and **16** (3*R*,4*R*,2′*R*) were obtained as single isomers after base-promoted cyclization of the corresponding 2″*R*-chloropanoyl derivatives, **11** (α*S*,2′*S*,2″*R*), and **12** (α*S*,2′*R*,2″*R*). As expected, enantiomeric pairs (**13**/**16** and **14**/**15**) show similar values of optical rotation, but of opposite sign. As KPs are not possible in this case, the yield of β-lactams is in general higher than those obtained for the corresponding 2′-benzyloxycarbonyl derivatives [41], but some hydrolysis of the benzyl ester was observed during the cyclization step workup (higher for 2′*R*-diastereoisomers compared to 2′*S*-counterparts).

### 2.2. Biological Activity

#### 2.2.1. Ca^2+^ Intracellular Influx Assay

**Figure A1:**
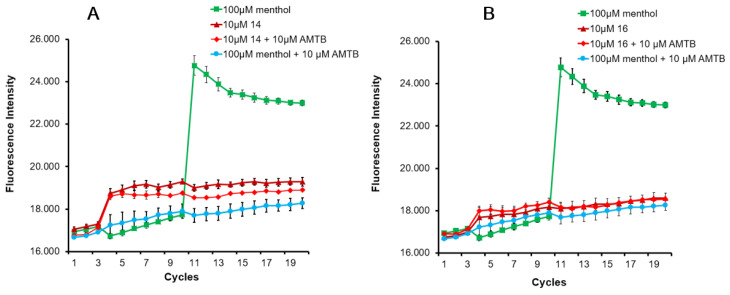
Effect of compounds on TRPM8 activity measured by fluorometric methods. Representative graph of compound 14 (**A**) and 16 (**B**) at 10 μM on HEK-rTRPM8 cells. After 3 basal fluorescence measurements compounds 14 (**A**) and 16 (**B**) were applied (maroon trace) with hardly any effect. An amount of 10 μM N-(3-aminopropyl)-2-[(3-methylphenyl)methoxy]-N-(2-thienylmethyl)-benzamide (AMTB) as a negative control was also applied in cycle 3 (blue trace). Subsequently after cycle 10, 100 μM menthol was added being the fluorescent signal completely blocked. An amount of 100μM menthol was also applied in absence of blockers as a positive control (green trace). An amount of 10 μM AMTB was added together with 14 and 16 (red trace) to ensure that the small increase in fluoresce after compounds’ addition was not due to any agonist activity.

The four obtained diastereomeric compounds were evaluated for their ability to inhibit menthol-induced Ca^2+^ intracellular influx into the cytosol on HEK293 cells heterologously expressing rat TRPM8. The well-known TRPM8 antagonist *N*-(3-aminopropyl)-2-[(3-methylphenyl)methoxy]-*N*-(2-thienylmethyl)-benzamide (AMTB) was used as control [45,46]. The results are depicted in Table 1. Some representative graphs for compound **14** and **16** at 10 μM on HEK-rTRPM8 cells are included in Figure A1.

In the Ca^2+^ fluorometry assay, all diastereoisomeric compounds were able to antagonize the menthol-induced activation of TRPM8 channels, but with different potencies. Thus, β-lactam derivatives of 3*S*,4*S* configuration (**13** and **15**) display micromolar and submicromolar antagonist activity, respectively. Their IC_50_ values are comparable to those found for stereoequivalent, enantiopure *N*-benzyloxycarbonyl derivatives previously described [41], thus indicating that the Z-group can be substituted by an *N*-dibenzyl moiety without significant changes in the TRPM8 activity. Comparing **13** to **15**, it is clear that the 2’*R*-stereogenic center in compound **15** results in one order of magnitude higher TRPM8 antagonist potency than the corresponding 2’*S*-isomer **13**. All *S*-configured compound **13** is equipotent to AMTB, while diastereoisomer **15** shows one order of magnitude higher IC_50_ value than the model antagonist.

We could also prepare enantiopure 3*R*,4*R* β-lactam diastereoisomeric compounds, a configuration not explored already within this family of compounds. 3*R*,4*R*-Configured β-lactams **14** and **16** are the most potent TRPM8 antagonists in this series, showing IC_50_ values of 0.3 and 0.02 μM. Once again, comparison between both configurations at 2’-position indicated that a 2´*R*-stereogenic center is preferred for high TRPM8 antagonist activity, with one order of magnitude higher potency for **16** *versus* **14**. Both compounds are more potent than the model antagonist AMTB, with isomer **16** showing two-orders of magnitude higher activity.

The dependence of the TRPM8 activity, activation or blocking, on the absolute configuration of the ligands has also been recorded in the literature for other families of TRPM8 agonists and antagonists [33,47,48]. Thus, a *N*,*N*-dibenzyl-D-Trp derivative showed highly reduced antagonist potency compared to the corresponding L-enantiomer [33]. Similarly, in a family of *N*-(thiophen-2-ylmethyl)-2-acetamide derivatives acting as TRPM8 agonists, the change of a unique chiral center provides either a nanomolar active or an inactive compound [47]. Approximately one order of magnitude reduced potency was also obtained for 4*S*-(+)-4-hydroxy-3,4-dihydrospiro[chromene-2,4′-piperidine] derivatives compared to the corresponding 4*R*-(-) analogues [48]. In symetrical tetrahydroisoquinoline derivatives, only a *trans*-disposition between C-1 and C-1′-substituents led to compounds with significant TRPM8 antagonist activity [30]. Similarly, in a recently described family of *N*-acyl-N-indanyl-α-phenylglycinamides derivatives, α-R isomers are from one to three orders of magnitude more potent than the corresponding S-diastereoisomers [49].

Here, we discovered that for β–lactams of general structure **II**, the 3*R*,4*R*-arrangement is preferred over the 3*S*,4*S*-configuration, and the results recognize a predilection for a 2′*R* stereocenter at the phenylalaninol-derived moiety. Within phenylalanine-phenylalaninol-derived β–lactams, and until other diasteroisomers could be prepared in enantiopure form, the 3*R*,4*R*,2′*R* isomer can be considered the eutomer. In general, for the *SS* configured isomers at 3,4-positions of the β–lactam ring, the *N*-dibenzylphenylalaninol-Phe-derived β–lactams described here show similar potencies as *N*-Z-phenylalaninol previous analogues [41].

Because some molecular cross-recognition may occur within thermoTRP members [50], we evaluated the effect of compounds **14** and **16** on recombinantly expressed hTRPV1 and hTRPA1 channels. Data shown in Table 2 indicate that these β–lactam derivatives marginally cross-reacted with these channels, thus substantiating that they are potent and selective inhibitors of TRPM8.

#### 2.2.2. Patch–Clamp Experiments

**Figure 2:**
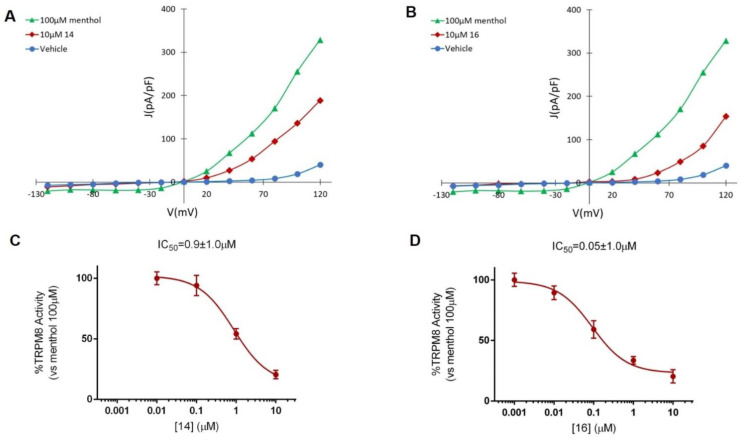
Compounds **14** and **16** block TRPM8-mediated responses evoked by menthol in rTRPM8-expressing HEK293 cells. (**A**,**C**). *I–V* curves obtained in HEK293 cells expressing TRPM8 and exposed to vehicle solution (vehicle; blue traces), 100 μM menthol (green trace), 100 μM menthol + 10 μM **14** (red trace) or to 100 μM menthol + 10 μM compound **16** (red trace). Peak current data were expressed as pA/pF (to facilitate comparison among cells of different sizes) and expressed as a function of antagonist concentrations. (**B**,**D**), Concentration/response curves for TRPM8 current blockade by compound **14** (**C**) or compound **16** (**D**) at a holding voltage of −60 mV. The solid lines represent fits of the experimental data to the following binding isotherm: y = max/(1 + x/IC50)n, where x is the drug concentration and n the Hill coefficient. The fitted values for IC_50_ were 0.9 ± 1.0 and 0.05 ± 1.0 for compound **14** and **16**, respectively. Each point is the mean ± SEM of 15 determinations, each obtained in different cells.

**Figure A2:**
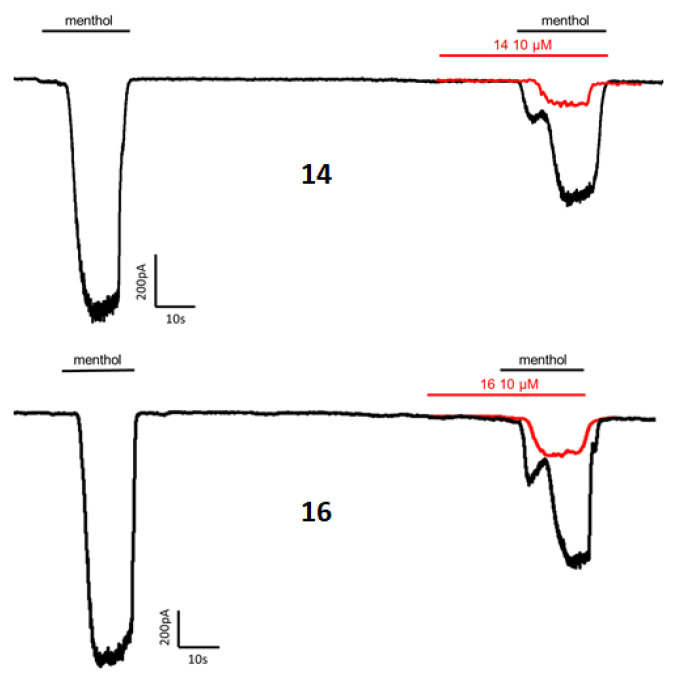
Compounds **14** and **16** inhibited TRPM8-mediated currents studied by whole cell Patch clamp. Representative menthol-evoked rTRPM8 current registered at a holding potential of -60 mV for control condition with two pulses of 100 μM menthol (black traces) and test condition with 30s perfusion of 10 μM **14** and **16** (red traces) before the second menthol pulse.

The TRPM8 antagonist activity of the most potent β–lactams in the previous Ca^2+^ intracellular influx test, **14** and **16**, was then confirmed by Patch-clamp experiments, using HEK293 cells expressing rat TRPM8 channels. In the electrophysiology assay, both compounds at 10 μM concentration were able to block TRPM8-mediated responses induced by menthol (Figure 2 and Figure A2, Table 3). As shown in Figure 2, perfusion with 100 μM menthol produces a strongly outward rectifying ionic current, described by the absence of current at negative potentials and the presence of a linear current increase (ohmic) at positive voltages ≥ 40 mV. After 10 μM of **14** was applied (Figure 2A, red), an important decrease on the menthol-induced TRPM8 activity at depolarizing voltages was observed. A similar behavior was detected after application of diastereomeric β–lactam **16** (Figure 2B). The dose–response curves for both compounds were obtained at a holding potential of −60 mV (Figure 2). The measured IC_50_ values were 0.9 ± 1.0 μM for **14** (Figure 2C) and 0.05 ± 1.3 μM for **16** (Figure 2D).

Electrophysiological results sustain that the 3*R*,4*R*-diastereoisomer **16** is more potent than the corresponding 3*S,*4*S*-isomer **14** blocking TRPM8 channel activity. Compound **16**, with an IC_50_ value of 50 nM is the most potent β–lactam derivative described to date within this family of TRPM8 antagonists. Its inhibitory activity of menthol-induced TRPM8 activation is comparable to many other structurally unrelated TRPM8 antagonists [28], although lower than that of *N*-dibenzyl-Trp-OMe [33].

**Figure 3:**
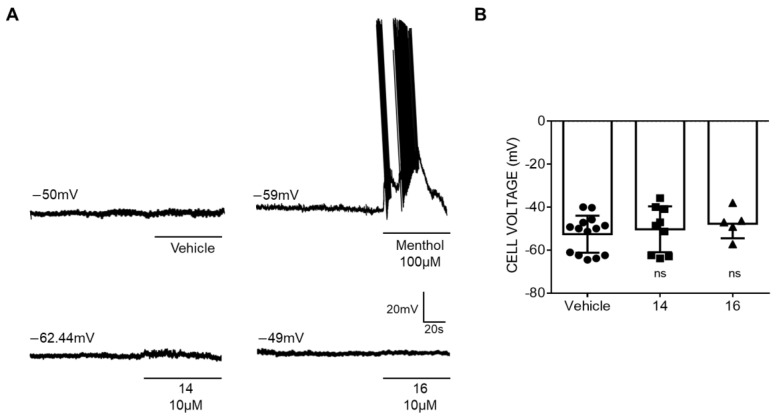
Compounds **14** and **16** do not affect the membrane resting potential of sensory neurons. (**A**) Representatives recordings of resting membrane potential measured under current-clamp configuration. The upper traces show the action potentials (APs) firings from the dorsal root ganglion (DRG) neuron elicited by 100 μM menthol and the lower traces show the voltage membrane in the presence of the β-lactams derivatives. (**B**) Changes in Vm in the absence and in the presence of 10 μM of compound **14** or **16** were analyzed by t-test, ns—no significance. Compounds were used at 10 μM. Data are given as means ± SEM; n ≥ 10 cells.

Next, we investigated the effect of the membrane of rat DRG sensory neurons. Under current-clamp, small sensory neurons displayed an resting membrane potential of −50 mV (Figure 3A, top trace, and Figure 3B). Exposure of the neurons to 10 μM of the β –lactam derivatives did not alter RMP of primary sensory neurons (Figure 3A, bottom traces and Figure 3B). Sensory neurons were functional as evidenced by action potentials fired exposed to 100 μM menthol (Figure 3A, top trace).

#### 2.2.3. Microelectrode Array Experiments

**Figure 4:**
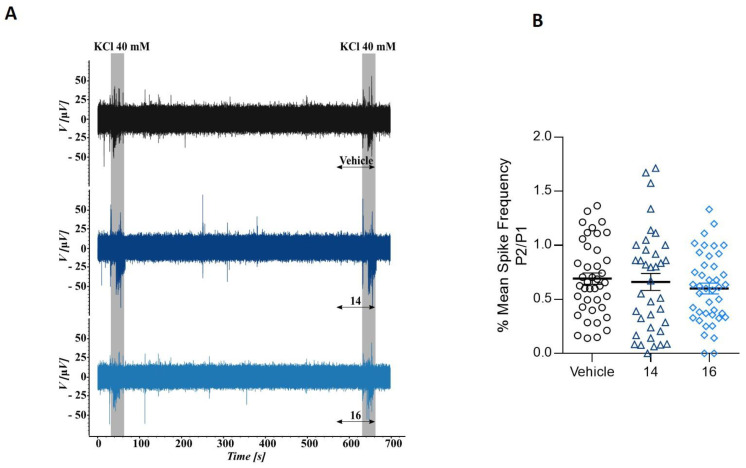
Compounds **14** and **16** do not alter potassium-evoked action potentials in primary cultures of rat sensory neurons. (**A**) Representative recordings of evoked action potentials in rat DRG neurons with 40 mM KCl (K) in the absence (top) and presence of 10 μM compound **14** (medium trace) or 10 μM compound **16** (bottom trace). (**B**) Mean spike frequency measured on the first pulse (P1) and second pulse (P2) of 40 mM KCl in absence or presence of 10 μM compounds.

To further interrogate β–lactam derivatives’ action on the excitability of DRG neurons we next used microelectrode arrays (MEA). This technology records the electrogenic activity of neural populations that are located near to an electrode, but it cannot differentiate the number of neurons or other cells contributing to it. We used MEA chips with 60 electrodes and monitored the electrical activity at each electrode to measure if the potent inhibitory effect of β–lactam derivatives act on voltage-gated Na^+^ and K^+^ channels present in neurons and responsible for action potential propagation. Neurons isolated from neonatal rat dorsal root ganglions (DRG) were cultured on MEA chambers. The experimental paradigm consisted in applying a first 15-s pulse (P1) of 40 mM KCl, to evoke action potentials. After a recovery period of 10 min, a second pulse (P2) of K^+^ in absence (vehicle, V) or presence of 10 μM compound **14** or compound **16** was applied to measure the effect of the compounds in K^+^-induced electrical excitability of nociceptors. Figure 4 shows a typical MEA recording from one electrode (upper trace). It can be observed the KCl evoked bursts of neural action potential firing. The application of 10 μM β–lactam derivatives **14** and **16** did not affected the electrogenicity induced by K^+^ (Figure 4A, middle and lower traces). These results suggest that the β–lactam derivatives **14** and **16** did not affect Kv and Nav channels, discarding a potential anesthetic effect, and further strengthening the channel selectivity of the compounds.

### 2.3. Molecular Modeling Studies

As for previous analogues [41], the fact that all isomers described here could antagonize TRPM8 receptors suggests that the binding of these diastereomeric compounds to the channel should occur in a pocket or area wide enough to accommodate different three-dimensional arrangements of the hydrophobic substituents. To identify possible binding pockets for these β-lactams in the TRPM8 channel, docking studies were performed with **13**–**16** diastereomeric compounds. Docking simulations were performed with Yasara software [51,52], in a model of a rat TRPM8 channel, generated from the cryo-electron microscopy (cryo-EM) structure of *Ficedula albicollis* TRPM8 (*fa*TRPM8, PDB code 6BPQ) [37]. Results from these studies indicated that all diastereoisomers preferentialy or exclusively bind to the pore zone, occupying up to four different subsites (Table 4). As for *N*-benzyloxycarbonyl-described analogues [41], the most populated subsite 1 is situated at the middle of the transmembrane region and involves TM5 (S5) and TM6 (S6) of one protein monomer and S6 of a second monomer. Subsite 2 is located at the internal mouth of the pore and is formed by the loops connecting TM6 and TRP domains of the four monomers forming the channel. Subsite 3 is located upper in the pore region, and involves residues in the S3, S4 transmembranes of one protomer and S6 of an adjacent subunit. Finally, subsite 4 corresponds to the external part of the pore and is delineated by the loops connecting S5 and S6 of three contiguous protomers. In all cases, most connections between the channel and the isomeric β-lactams are Van de Waals interactions (VdW) and π–π stacking.

**Figure 5:**
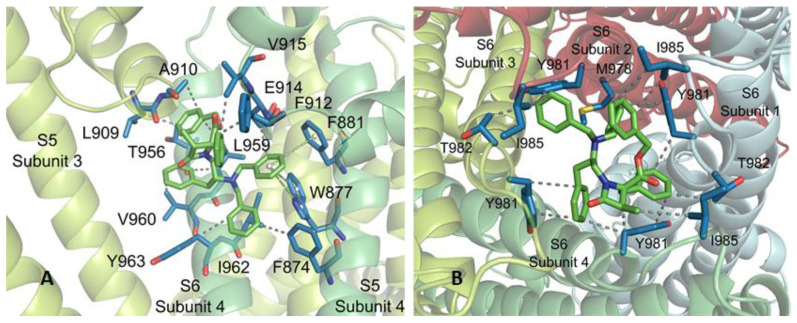
Subsites 1 (**A**) and 2 (**B**) found for β–lactam **16** (carbon atoms in green). WdV interactions are indicated by doted gray lines, and π–π stacking by centroids and non-continuous pale green lines. Channel residues involved in the interactions are shown in blue and labelled.

**Figure A3:**
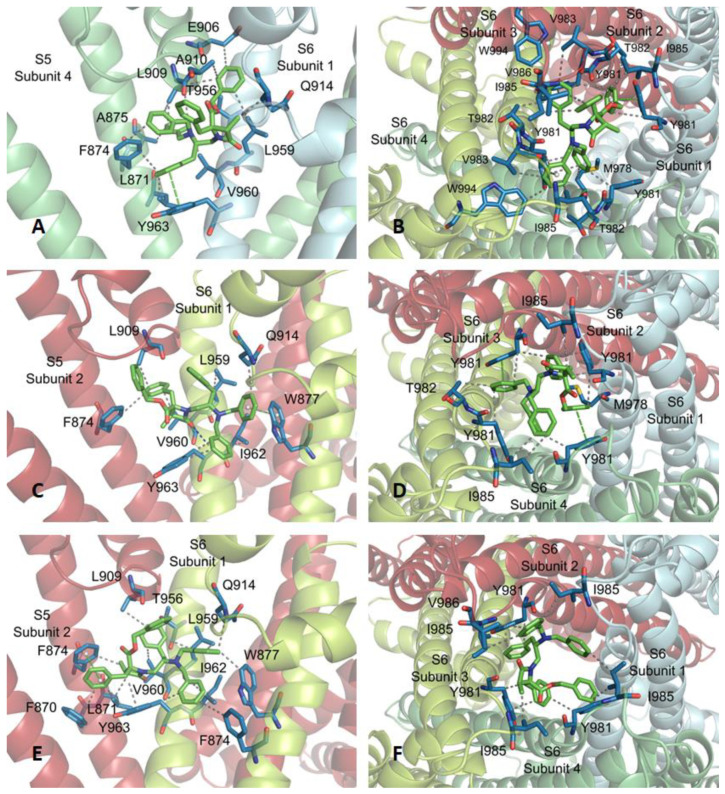
Subsite 1 (**A**) and 2 (**B**) found for β-lactam **13**. Subsites 1 (**C**) and 2 (**D**) found for β-lactam **14**. Subsite 1 (**E**) and 2 (**F**) found for β-lactam **15**. WdV interactions are indicated by dotted gray lines, and π–π stacking by centroids and non-continuous pale green lines. Channel residues involved in the interactions are shown in blue and labelled.

At subsite 1, 3*R*,4*R*,2′*R*-β-lactam **16** interconnects, on one hand, with a subunit of the channel through F874, W877 and F881, situated in one face of S5 helix, F912, E914 and V915, within the S5-S6 linker, and with T956, L959, V960, I962 and Y963 side-chains of TM S6 (Figure 5). On the other hand, the complex structure is stabilized by interaction with L909 and A910 of the S5-S6 connecting segment of an adjacent subunit. Among these interactions, those with W877 and F881 correspond to T-shaped π–π stackings with the phenyl ring of one of the *N*-benzyl substituents. Quite similar interconnections were found for diastereoisomers **13**–**15** at subsite 1, showing repeated VdW intermolecular forces with F874, L909 and L959, and one or two π–π stackings (Figure A3). The interplay between compound **16** and the TRPM8 channel at subsite 2 is mediated by VdW interactions with hydrophobic residues at the cytosolic area of the pore, namely M978 (one subunit), Y981 (all subunits), T982 (two subunits) and I985 (three subunits). Related attachments of compounds **13**–**15** to this subsite were observed, but compound **14** revealed three additional π–π stackings with channel Y981 residues.

**Figure A4:**
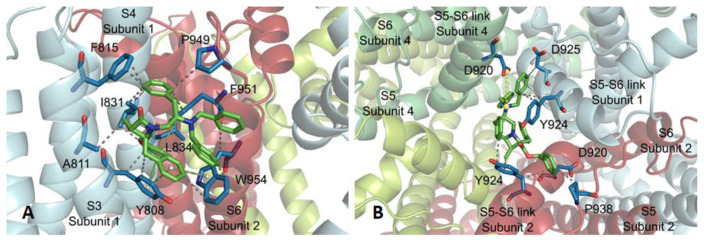
Subsite 3 (**A**) for β-lactam **14** and subsite 4 for β-lactam **16** (**B**). WdV interactions are indicated by dotted gray lines, and π–π stacking by centroids and non-continuous pale green lines. Channel residues involved in the interactions are shown in blue and labelled.

To exemplify the binding at subsite 3, the complex between compound **14** and the TRPM8 channel is shown in Figure A4. The main hydrophobic contacts are with S3 (Y808, A811, F815) and S4 (I831, L834) transmembrane segments of one protomer, and an S6 residue (W945) of a contiguous subunit. Y808, F951, and W954 aromatic side-chains in the receptor are involved in T- type π–π interactions with the phenyl groups of 4-Bn and OBn moieties of the ligand. Subsite 4 is defined by the loops joining S5–S6, including the pore helix, of three contiguous protomers. In this subsite, compound **16** locates above the pore helix, and interacts with Y924 (from two subunits), D920 and D925 from subunits 1 and 2, and forms a salt bridge between the *N*-tertiary amine of the ligand and the D920 carboxylate of subunit 4. Very close interactions were observed for β–lactams **13**–**15** at subsites 3 and 4 (not shown).

The putative sites for our β-lactams at the TRPM8 are different to that described for the model TRPM8 antagonist AMTB. Cryo-electron microscopy has demonstrated that AMTB accommodates in a hole delimited by the lower half of S1-S4 transmembrane domain and the TRP domain [53]. This adaptable binding pocket also lodges the TC-I antagonist [53], although in a different pose than AMTB, a menthol-derived agonist [39], and was suggested by molecular modeling studies on tryptophan-derived antagonists [33]. The different behavior of our molecules could be due to their large size, and the interaction at the upper extracellular part of the channel (subsite 4) could initiate their ulterior distribution through the pore region (subsites 1–3).

## 3. Materials and Methods

Details on the preparation of suitable synthetic intermediates in the road to the described β–lactams form commercially available Phe and phenilalaninol derivatives are given in Appendix A.

### 3.1. β-Lactam Formation

A solution of the corresponding *N*-alkyl-*N*-chloropropionyl-Phe-OBn derivative (1.6 mmol) in dry CH_3_CN (4 mL), under Ar atmosphere, was treated with BTPP (2.4 mmol, 0.75 mL) and stirred at rt until consumption of the starting material. After removal of the solvent, the resulting residue was extracted with EtOAc, and successively washed with 0.1 M HCl, H_2_O and brine. The organic layer was separated, dried over Na_2_SO_4_, filtered, and concentrated. The resulting residue was purified by flash chromatography on silica gel, using the indicated eluent system.

#### 3.1.1. 4*S*-Benzyl-1-[(2′*S*-dibenzylamino-3′-phenyl-)prop-1′-yl]-4-benzyloxicarbonyl-3*S*-methyl-2-oxoazetidine (**13**)

Syrup. Yield: 63% (from **9**). Eluent: EtOAc:Hex (1:4). HPLC: tR = 7.19 min. [α]_D_ = +49.16 (c 1, CHCl_3_). ^1^H NMR (400 MHz, CDCl_3_): δ 7.40–6.89 (m, 25H, Ar), 5.23 (d, 1H, *J* = 11.9 Hz, OCH_2_), 5.01 (d, 1H, *J* = 11.9 Hz, OCH_2_), 3.64 (d, 2H, *J* = 13.9 Hz, NCH_2_), 3.49 (m, 1H, 2’-H), 3.44 (d, 2H, *J* = 13.9 Hz, NCH_2_), 3.41 (dd, 1H, *J* = 13.9, 4.0 Hz, 1’-H), 3.22 (dd, 1H, *J* = 13.8, 9.8 Hz, 1’-H), 3.10 (q, 1H, *J* = 7.5 Hz, 3-H), 2.99 (d, 1H, *J* = 14.2 Hz, 4-CH_2_), 2.93 (d, 1H, *J* = 14.2 Hz, 4-CH_2_), 2.86 (dd, 1H, *J* = 14.4, 5.0 Hz, 3’-H), 2.75 (dd, 1H, *J* = 14.4, 9.1 Hz, 3’-H), 1.07 (d, 3H, *J* = 7.5 Hz, CH_3_). ^13^C NMR (75 MHz, CDCl_3_): δ 170.8 (COO), 169.1 (C2), 140.4, 139.7, 135.2, 135.0, 130.0, 129.6, 129.2, 128.9, 128.8, 128.7, 128.6, 128.2, 128.1, 127.3, 126.9, 125.9 (Ar), 68.4 (OCH_2_), 67.2 (C4), 57.9 (C2’), 57.8 (C3), 53.3 (NCH_2_), 43.0 (C1’), 40.9 (4-CH_2_), 36.2 (C3’), 10.6 (CH_3_). MS (ES)^+^: 623.01 [M+H]^+^. Exact Mass calculated for C_42_H_42_N_2_O_3_: 622.31954; found: 622.31993.

#### 3.1.2. 4*S*-Benzyl-1-[(2′*R*-dibenzylamino-3′-phenyl)prop-1′-yl]-4-benzyloxicarbonyl-3S-methyl-2-oxoazetidine (**14**)

Syrup. Yield: 49% (from **10**). Eluents: EtOAc:Hex (1:6). HPLC: t_R_ = 7.29 min (gradient of 5% to 100% of A, in 20 min). [α]_D_ = +122.29 (c 0.24, CHCl_3_). HPLC: t_R_ = 7.29 min (gradient of 5% to 100% of A, in 20 min). ^1^H NMR (400 MHz, CDCl_3_): δ 7.36–6.93 (m, 25H, Ar), 5.06 (d, 1H, *J* = 12.0 Hz, OCH_2_), 4.84 (d, 1H, *J* = 12.0 Hz, OCH_2_), 3.56 (d, 2H, *J* = 13.9 Hz, NCH_2_), 3.47 (d, 2H, *J* = 13.9 Hz, NCH_2_), 3.46 (m, 1H, 3-H), 3.37 (m, 1H, 2′-H), 3.14 (dd, 1H, *J* = 14.2, 8.1 Hz, 1′-H), 3.08 (m, 2H, 3’-H, 4-CH_2_), 2.81 (m, 3H, 4-CH_2_, 1′-H, 3′-H), 1.00 (d, 3H, *J* = 7.5 Hz, CH_3_). ^13^C NMR (75 MHz, CDCl_3_): δ 170.9 (COO), 169.9 (C2), 140.3, 140.0, 135.5, 135.1, 129.9, 129.8, 129.7, 129.1, 129.0, 128.8, 128.75, 128.7, 128.2, 127.4, 126.9, 126.0 (Ar), 68.6 (C4), 67.3 (OCH_2_), 58.7 (C2’), 54.4 (C3), 53.2 (NCH_2_), 42.1 (C1’), 40.6 (4-CH_2_), 35.9 (C3’), 10.5 (CH_3_). MS (ES)^+^: 623.52 [M+H]^+^. Exact Mass calculated for C_42_H_42_N_2_O_3_: 622.31954; found: 622.31861.

#### 3.1.3. 4*R*-Benzyl-1-[(2′*S*-dibenzylamino-3′-phenyl)prop-1′-yl]-4-benzyloxicarbonyl- 3R-methyl-2-oxoazetidine (**15**)

Syrup. Yield: 57% (from **11**). Eluent: EtOAc:Hex (1:4). HPLC: t_R_ = 7.29 min. [α]_D_ = −122.76 (c 0.26, CHCl_3_). ^1^H NMR (400 MHz, CDCl_3_) and ^13^C NMR (75 MHz, CDCl_3_): δ identical to its enantiomer **14**. MS (ES)^+^: 622.95 [M+H]^+^. Exact Mass calculated for C_42_H_42_N_2_O_3_: 622.31954; found: 622.32219.

#### 3.1.4. 4*R*-Benzyl-1-[(2′*R*-dibenzylamino-3′-phenyl-)prop-1′-yl]-4-benzyloxicarbonyl- 3R-methyl-2-oxoazetidine (**16**)

Syrup. Yield: 38% (from **12**). Eluents: EtOAc:Hex (1:6). HPLC: t_R_ = 7.19 min (gradient of 5% to 100% of A, in 20 min). [α]_D_ = −49.09 (c 0.23, CHCl_3_). ^1^H NMR (400 MHz, CDCl_3_) and ^13^C NMR (75 MHz, CDCl_3_): δ identical to its enantiomer **13**. MS (ES)^+^: 623.52 [M+H]^+^. Exact Mass calculated for C_42_H_42_N_2_O_3_: 622.31954; found: 622.32026.

### 3.2. Functional Assays by Calcium Microfluorimetry

Compounds were evaluated for their activity against rTRPM8 using microfluorometry based calcium flux assays with Fluo-4 NW Ca^2+^ dye and fluorescence were measured at excitation wavelength of 485 nm and emission wavelength of 520 nm using POLASTAR plate reader (BMG Labtech) [40]. Briefly human embryonic kidney cell line (HEK) stably transfected with rTRPM8 were seeded in 96-well plates at a cell density of 30,000 cells and 2 days before the medium was replaced with 100 μL of the dye loading solution Fluo-4 NW supplemented with probenecid 2.5 mM. After 1 h incubation at 37 °C, plates were transferred to the plate reader and the baseline fluorescence was recorded for 3 cycles before the addition of vehicle, compound at different concentrations and the antagonist, 10 μM AMTB for TRPM8. Fluorescence intensity was recorded during 7 more cycles and the agonist was added, 100 μM menthol. Fluorescence intensity was recorded during 10 more cycles.

Data analysis: the fluorescence values obtained for each compound concentration were normalized to that prompted by the control agonist (100 μM menthol). Decrease in menthol signal was expressed as percentage of inhibition (%). All data are expressed as the mean ± standard deviation (SD). Each condition was assessed in triplicate (n = 3) in 3 independent experiments (N = 3). The Z-factor was calculated in each assay using the following equation: (3*(SDmax + SDmin))/(Mean max-Mean min). In all the experiments, the Z-factor was ≥ 0.5. To calculate IC_50_, normalized responses (%) versus log (μM) were adjusted to a non-linear fit with variable slope, a four-parameter dose–response curve following curve Y = 100/(1 + 10^((Log IC_50_-X) × HillSlope)) where X = % normalized response and Y= log (μM).

### 3.3. Functional Assays by Patch-Clamp Electrophysiology

Whole-cell patch-clamp recordings from HEK-rTRPM8 cells were carried out 2 days after seeding on 12-mm Ø glass coverslips treated with poly-L-lysine solution (Sigma Aldrich, Spain) [40]; the intracellular pipette solution contained (in mM) 150 NaCl, 5 EGTA, 3 MgCl_2_ and 10 HEPES, adjusted to pH 7.2 with NaOH, and the extracellular solution contained (in mM) 150 NaCl, 6 CsCl, 1.5 CaCl_2_, 1 MgCl_2_, 10 D-glucose and 10 HEPES, adjusted to pH 7.4 with NaOH. The TRPM8 activity was measured by the application of two pulses of 100 μM menthol in a time interval of of 2 min and a 30-s perfusion of the different compound concentrations before the second menthol pulse.

Current-Clamp recordings from rat DRGs were carried out 24–48 h after seeding on 12-mm Ø glass coverslips treated with poly-L-lysine solution and Laminin (Sigma Aldrich). The intracellular pipette solution contained (in mM): 4 NaCl, 126 K gluconate, 0.02 CaCl2, 1 MgSO4, 5 HEPES, 15 glucose, 3 ATP, 0.1 GTP and 5 EGTA, pH 7.2 with KOH. The extracellular solution contained (in mM): 140 NaCl, 4 KCl, 2 CaCl2, 2 MgCl2, 10 HEPES, 5 glucose and 20 mannitol, pH 7.4 with NaOH. The membrane potential was measured in the absence and the presence of compounds 14 and 16 at 10 μM in a time interval of 1 min.

Data were sampled at 10 kHz (EPC10 amplifier with PatchMaster 2.53 software, HEKA Electronics, Lambrecht, Germany) and low-pass filtered at 3 kHz for analysis (PatchMaster 2.53 and GraphPad Prism 5, Graphpad Software, USA). The series resistance was <10 MΩ and to minimize voltage errors was compensated to 60–80%. All measurements were performed at 24–25 °C. Cell capacitance was measured and used to estimate the current density (J, pA/pF).

Data analysis: Results are expressed as the percentage of remaining activation of the TRPM8 channel. This is calculated by normalizing the ratio (p2/p1) of testing conditions to the ratio (p2/p1) of the control condition. Analysis of the data was performed by GraphPad 6.0, the Ordinary One-Way ANOVA analysis followed by the post hoc Bonferroni test stablished multiple comparisons and the ROUT method (Q = 10%) identified data outliers. A non-linear regression curve and IC_50_ were obtained by the representation of log (inhibitor) vs. response. All data are expressed as the mean ± standard error of the mean (SEM) (n = 5–8).

### 3.4. Functional Assays by Microelectrode Arrays

Extracellular recordings were performed as described in Nikolaeva-Koleva et al. [54], Briefly, measurement of neuronal firing activity was performed by applying two short 15-s applications (defined as P1 and P2, respectively) of the stimulus (KCl 40 mM) using a continuous perfusion system (2 mL/min). Between each stimulus, cells were washed with external solution for 10 min. Treated cells were perfused with β–lactam derivatives) 1 min before and together with 40 mM KCl. All measurements were performed at ~34.5 °C (Multichannel Systems Temperature Controller).

Data analysis: Data were analyzed using an MC_RACK spike sorter with a sample rate of 25 kHz and a Butterworth high-pass 2nd order filter applied with 200 Hz cutoff. An evoked spike was defined when the amplitude of the neuronal electrical activity was established by automatic threshold estimation at −5.0 μV Std. Dev. Spiking activity was measured in a temporal interval of 30 s, starting right after instillation of the activating stimuli. Electrodes not displaying electrical activity in the first KCl pulse were discarded. The recorded signals were then processed to extract mean spike frequency for each pulse (P1–P2). Then, the ratio P2/P1 of mean spike frequency was calculated and normalized to vehicle for comparing the different conditions used.

### 3.5. Docking Studies

These studies were performed as previously described [41]. Briefly, starting from the cryo-EM structure of *Ficedula albicollis* TRPM8 [37], the missing loops were added by using Yasara [51,55]. Then, the rat TRPM8 sequence (Uniprot Q8R455) was modeled on completed *Ficedula a.* structure. Sequence alignment between rat and *Ficedula a.* TRPM8 was performed with ClustalO [56], and homology modelling with the standard protocol implemented in Yasara (version 19.9.17) [51,52]. Global docking was accomplished with AutoDock [57] implemented in Yasara, and consisted in a total of 800 flexible docking runs that were then set and clustered around the putative binding sites, following by a simulated annealing optimization of each generated complex using an Assisted Model Building with Energy Refinement (AMBER03) force field [58]. The best binding energy complex in each cluster was stored, analyzed, and used to select the best orientation of the interacting partners.

Visualization and edition of the molecules were also performed with Yasara (http://www.yasara.org (accessed on: 12 December 2020)). Finally, figures were drawn with the open source Pymol (The PyMOL Molecular Graphics System, Version 1.8 Schrödinger, LLC, New York, NY, USA) obtained at http://www.pymol.org (accessed on: 12 December 2020).

## 4. Conclusions

Since interactions of drugs with macromolecules (receptors, enzymes, channels, DNA) have long been recognized to occur in a stereoselective manner, it is important to know how the absolute configuration of a given compound correlates to its pharmacodynamic properties. To date, in our series of β–lactam TRPM8 antagonists we have mainly studied all S stereoisomers. Here, we evaluated and compared to each other, four synthetically accessible diastereoisomers of a β–lactam obtained from enantiopure *N*-dibenzylphenylalaninol-Phe conjugates. The *N*-dibenzylamino group can replace hydrophobic urethane moieties at 2′-position moieties without significant changes in the TRPM8 antagonist activity, but avoiding the formation of yield-lowering 2-ketopyperazine regioisomeric compounds [41]. The previously non-explored 3*R*,4*R*-configuration at the β–lactam ring, resulted in compounds that, at least, are one order of magnitude more potent than the corresponding 3*S*,4*S* enantiomeric analogues. At the penylalaninol-derived moiety, the results descibed here corroborate the importance of an *R* configuration at the 2′ position for potent TRPM8 antagonist activity, with 2’*R*-isomers clearly preferred over their 2′*S*-equivalents. While other enantiopure diasteroisomers could not be prepared, the 3R,4R,2′R isomer should be considered the eutomer of this family of phenylalanine-phenylalaninol-derived β–lactams. Docking studies with these diastereomeric compounds strongly suggest that they interact by the TRPM8 pore zone, with possible binding pockets different from that described for AMTB and other channel modulators. Our results open up new opportunities for isomerically pure TRPM8 antagonists, through the synthesis and biological study of innovative analogues within this family of β–lactams, which are currently being investigated in our labs and will be published in due course.

## Figures and Tables

**Table 1:** Activity at TRPM8 channels of β–lactam derivatives (Ca^2+^ influx assay).

Compoundd	Configuration	% Blockade 50 μM	% Blockade 5 μM	IC_50_ (μM)	95% Confidence Intervals
13	3*S*,4*S*,2′*S*	86.1 ± 5.0	57.7 ± 7.2	3.1 ± 1.1	2.574 to 3.997
14	3*S*,4*S*,2′*R*	87.7 ± 3.1	78.9 ± 1.7	0.3 ± 1.0	0.2162 to 0.2804
15	3*R*,4*R*,2′*S*	90.3 ± 2.8	71.1 ± 4.3	0.7 ± 1.1	0.6376 to 0.9071
16	3*R*,4*R*,2′*R*	98.5 ± 3.2	87.3 ± 8.1	0.02 ± 1.1	0.010 to 0.019
1 ^a^	3*S*,4*S*,2′*S*			2.4 ± 1.2	
AMTB ^a^	*_*			7.3 ± 1.5	

^a^ Values taken from reference [41].

**Table 2:** Activity at TRPV1 and TRPA1 channels of β–lactam derivatives (Ca^2+^ influx assay).

Compoundd	Configuration	% TRPV1 Blockade at 50 μM	% TRPA1 Blockade at 50 μM
13	3*S*,4*S*,2′*S*	7.9 ± 3.2	3.7 ± 4.1
14	3*S*,4*S*,2′*R*	2.8 ± 4.1	2.4 ± 1.4
15	3*R*,4*R*,2′*S*	5.3 ± 3.4	2.6 ± 1.9
16	3*R*,4*R*,2′*R*	4.2 ± 3.2	0.5± 4.1

**Table 3:** Patch-clamp experiments (whole cell configuration in HEK293 cells expressing TRPM8 channels).

Compound	Configuration	% Blockade at 10 μM	IC_50_ (μM)
14	3*S*,4*S*,2′*R*	77.2 ± 2.7	0.9 ± 1.0
16	3*R*,4*R*,2′*R*	79.3 ± 2.7	0.05 ± 1.0
1	3*S*,4*S*,2′*S*		1.4 ± 1.1

**Table 4:** Main sites found for compounds **13**–**16**, and statistical distribution of the docking solutions.

Subsite	Location	% of Docking Solutions (Estimated Binding Energies, kcal/mol)			
		13	14	15	16
1	Inner pore, S5S6, S6	40% (10.64)	42% (10.53)	50% (10.58)	52% (10.26)
2	Internal mouth pore	34% (11.44)	18% (10.15)	22% (10.14)	20% (11.40)
3	Pore, high S3-S4, S6	12% (9.95)	24% (10.24)	10% (9.24)	12% (10.32)
4	Pore, external tower	4% (10.20)	16% (10.43)	18% (11.46)	16% (10.41)

## Data Availability

Not applicable.

## Abbreviations

AMTB	*N*-(3-Aminopropyl)-2-[(3-methylphenyl)methoxy]-*N*-(2-thienylmethyl)benzamide
Bn	Benzyl
BTPP	Phosphazene base P_1_-t-Bu-tris(tetramethylene)
HEK	Human embryo kidney cells
HPLC	High-performance liquid chromatography
KP	2-Ketopiperazine
NMR	Nuclear magnetic resonance
Ns	Nosyl
Phe	Phenylalanine
PIP2	Phosphatidylinositol bisphosphate
SD	Standard deviation
TRP	Transient receptor potential channel
TRPM8	Transient receptor potential melastatin type 8 channel
Z	Benzyloxycarbonyl

## Appendix A

### Appendix A.1. Chemistry, General Procedures

Reaction monitoring: TLC silica gel plates (Merck 60 F254, Spain) and analytic HPLC (Agilent Technologies 1120 Compact LC, Spain), Eclipse C18, 4.6 × 150 mm, 5 μm, reversed-phase column; mobile phase (A:B), CH_3_CN(A)/H_2_O(0.05% TFA)(B); flux, 1.5 mL/min; detection, UV, 254 nm). Chromatographic separations: flash column, silica gel Merck 60 (230–400). Optical rotations were measured in a Perkin Elmer 141 polarimetter. ^1^H NMR spectra: Varian INOVA-300 (300 MHz), Bruker 300 (300 MHz), and Varian INOVA-400 (400 MHz), with TMS as internal standard. ^13^C NMR spectra: INOVA-300 (75 MHz) and Bruker 300 (75 MHz). Chemical shifts are expressed in ppm, the coupling constants are expressed in Hz. Mass spectra; electrospray, positive mode, HPLC-MS Waters spectrometer. Exact mass: high resolution mass spectra (ESI-HRMS) were recorded on an Agilent 6520 Q-TOF instrument.

### Appendix A.2. Preparation of Synthetic Intermediates on the Road to Β-Lactam Derivatives

#### Appendix A.2.1. Synthesis of *N*-Ns-*N*-alkyl-Phe-OBn Derivatives

A solution of Ns-L-Phe-OBn (**2**, 3.8 mmol) in dry THF (33 mL) was treated with the corresponding alcohol derivative (**3** or **4**, 3.8 mmol) and PPh_3_ (3.8 mmol, 1 g). Then, the reaction mixture was treated with diisopropyl azodicarboxylate (DIAD) (3.8 mmol, 0.75 mL), under Ar atmosphere. The reaction mixture was stirred overnight at rt. After removal of the solvent in vacuum, the resulting residue was purified by flash chromatography on silica gel, using the indicated eluent system.

##### *N*-[(2′*S*-Dibenzylamino-3-phenyl-)prop-1′-yl]-Ns-L-Phe-OBn (**5**)

Syrup. Yield: 63% (from Ns-L-Phe-OBn (**2**) and **3**). Eluent: EtOAc:Hex (1:5). HPLC: t_R_= 18.40 min. ^1^H NMR (400 MHz, CDCl_3_): δ 7.52–6.77 (m, 29H, Ar), 4.66 (s, 2H, OCH_2_), 4.54 (dd, 1H, *J* = 9.7,5.5, α-Phe), 3.83 (dd, 1H, *J* = 14.8,5.7, 1-H), 3.68 (d, 2H, *J* = 13.7, NCH_2_), 3.53 (d, 2H, *J* = 13.7, NCH_2_), 3.32 (dd, 1H, *J* = 14.8,8.5, 1-H), 3.20 (m, 1H, 2-H), 2.81 (dd, 1H, *J* = 13.9, 8.5, 3-H), 2.67 (m, 3H, β-Phe and 3-H). ^13^C NMR (75 MHz, CDCl_3_): δ 169.6 (COO), 147.8, 139.8, 139.7, 136.4, 135.0, 134.0, 133.3, 131.9, 131.9, 129.7, 129.6, 129.4, 129.2, 128.6, 128.55, 128.5, 128.45, 128.4, 128.3, 128.25, 127.0, 126.9, 126.8, 126.2, 124.4 (Ar), 67.1 (OCH_2_), 62.0 (α-Phe), 57.8 (2-C), 53.2 (NCH_2_), 47.1 (C1), 36.8 (C3), 34.6 (β-Phe). MS (ES)^+^: 754.35 [M+H]^+^.

##### *N*-[(2′*R*-Dibenzylamino-3-phenyl-)prop-1′-yl]-Ns-L-Phe-OBn (**6**)

Syrup. Yield: 84% (from Ns-L-Phe-OBn (**2**) and **4**). Eluents: EtOAc:Hex (1:5). HPLC: t_R_ = 18.36 min (gradient of 5% to 100% of A, in 20 min). ^1^H NMR (400 MHz, CDCl_3_): δ 7.54–6.76 (m, 29H, Ar), 4.92 (d, 1H, *J* = 12.0 Hz, OCH_2_), 4.78 (m, 1H, α-Phe), 4.77 (d, 1H, *J* = 12.0 Hz, OCH_2_), 3.85 (dd, 1H, *J* = 15.0, 10.9 Hz, 1-H), 3.68 (d, 2H, *J* = 14.0 Hz, NCH_2_), 3.58 (dd, 1H, *J* = 15.0, 3.8 Hz, 1-H), 3.53 (d, 2H, *J* = 14.0 Hz, NCH_2_), 3.21 (m, 1H, 2-H), 3.08 (dd, 1H, *J* = 13.8, 9.5 Hz, 3-H), 2.90 (dd, 1H, *J* = 15.1, 4.4 Hz, β-Phe), 2.86 (dd, 1H, *J* = 13.8, 5.1 Hz, 3-H), 2.74 (dd, 1H, *J* = 15.1, 11.0 Hz, β-Phe). ^13^C NMR (75 MHz, CDCl_3_): δ 169.3 (COO), 148.2, 140.7, 139.7, 136.6, 134.8, 133.6, 131.7, 131.5, 129.8, 129.3, 128.9, 128.8, 128.7, 128.6, 128.3, 128.0, 127.0, 126.9 (Ar), 67.4 (OCH_2_), 61.9 (Cα-Phe), 57.9 (C2), 53.1 (NCH_2_), 47.4 (C1), 37.8 (C3), 35.3 (Cβ-Phe). MS (ES)^+^: 754.42 [M+H]^+^.

#### Appendix A.2.2. Removal of the Ns Group

A solution of the corresponding *N*-Ns-*N*-alkyl-Phe-OBn derivative (1.1 mmol) in CH_3_CN (20 mL) was treated with K_2_CO_3_ (3.3 mmol, 0.455 g). Then, thiophenol (2.2 mmol, 0.22 mL) was added to the mixture and the reaction was stirred overnight at rt. The solvent was removed in a vacuum and the resulting residue was extracted with AcOEt and washed successively with H_2_O and a saturated solution of NaCl. The organic phase was separated and dried over dry Na_2_SO_4_, filtered, and concentrated. The resulting residue was purified by flash chromatography on silica gel, using the indicated eluent system.

##### *N*-[(2′*S*-Dibenzylamino-3′-phenyl-)prop-1′-yl]-L-Phe-OBn (**7**)

Syrup. Yield: 77% (from **5**). Eluent: EtOAc:Hex (1:7). HPLC: t_R_ = 7.23 min. ^1^H NMR (400 MHz, CDCl_3_): δ 7.28–6.87 (m, 25H, Ar), 4.96 (s, 2H, OCH_2_), 3.60 (d, 2H, *J* = 13.7, NCH_2_), 3.43 (d, 2H, *J* = 13.7, NCH_2_), 3.35 (dd, 1H, *J* = 7.8, 5.9, α-Phe), 2.87 (m, 2H, 2’-H and 3’-H), 2.85 (dd, 1H, *J* = 13.5, 5.8, β-Phe), 2.73 (dd, 1H, *J* = 13.5, 7.8, β-Phe), 2.50 (m, 2H, 1-H, 1’-H), 2.38 (dd, 1H, *J* = 14.9,10.1, 3’-H). ^13^C NMR (75 MHz, CDCl_3_): δ 174.0 (COO), 140.2, 139.9, 137.6, 135.8, 129.5, 129.45, 129.4, 128.9, 128.8, 128.75, 128.65, 128.6, 128,5 128.4, 127.0, 126.9, 126.8, 1259 (Ar), 66.3 (OCH_2_), 63.2 (α-Phe), 59.8 (C2’), 53.7 (NCH_2_), 47.5 (C1’), 39.3 (β-Phe), 33.4 (C3’). MS (ES)^+^: 569.27 [M+H]^+^.

##### *N*-[(2′*R*-Dibenzylamino-3′-phenyl-)prop-1′-yl]-L-Phe-OBn (**8**)

Syrup. Yield: 99% (from **6**). Eluents: EtOAc:Hex (1:10). HPLC: t_R_ = 7.19 min (gradient of 5% to 100% of A, in 20 min). ^1^H NMR (400 MHz, CDCl_3_): δ 7.31–7.00 (m, 25H, Ar), 5.30 (s, 1H, NH), 5.02 (d, 1H, *J* = 12.0 Hz, OCH_2_), 4.98 (d, 1H, *J* = 12.0 Hz, OCH_2_), 3.78 (d, 2H, *J* = 13.3 Hz, NCH_2_), 3.46 (m, 1H, α-Phe), 3.36 (d, 2H, *J* = 13.3 Hz, NCH_2_), 3.10 (dd, 1H, *J* = 13.0, 3.7 Hz, 1′-H), 3.01 (m, 2H, 2′-H, β-Phe), 2.90 (dd, 1H, *J* = 13.4, 6.9 Hz,1′-H), 2.69 (m, 1H β-Phe), 2.29 (m, 2H, 3’-H). ^13^C NMR (75 MHz, CDCl_3_): δ 174.3 (COO), 140.1, 139.9, 136.9, 135.8, 129.8, 129.3, 129.2, 128.8, 128.5, 128.4, 128.35, 128.3, 127.0, 126.8, 126.0 (Ar), 66.3 (OCH_2_), 62.2 (Cα-Phe), 59.8 (C2′), 53.1 (NCH_2_), 47.3 (C1’), 39.9 (Cβ-Phe), 32.5 (C3′). MS (ES)^+^: 569.55 [M+H]^+^.

#### Appendix A.2.3. Synthesis of N-Alkyl-N-Chloropropionyl-Xaa Derivatives

First, a solution of (*R*)- or (*S*)-2-chloropropionic acid (0.126 mL, 1.47 mmol) and Cl_3_CCN (0.19 mL, 1.96 mmol) in THF (8 mL) was treated at 0ºC with PPh_3_ (0.513 g, 1.96 mmol), and the reaction mixture was stirred for 30 min. Then, a solution of the corresponding secondary amine (0.98 mmol) and propylene oxyde (1 mL, 14.7 mmol) in THF (2 mL) was added dropwise to the first reaction mixture. Stirring was continued for 48 h and the solvent was evaporated. The resulting residue was dissolved in Et_2_O, filtered over celite and concentrated under a vacuum. The resulting residue was purified by flash chromatography on silica gel, using the indicated eluent system.

##### *N*-(2″*S*-Chloropropanoyl)-*N*-[2′*S*-dibenzylamino)-3′-phenyl)prop-1′-yl]-L-Phe-OBn (**9**)

Syrup. Yield: 25% (from **7**). Eluent: EtOAc:Hex (1:6). Mixture of rotamers M,m = 2:1. HPLC: t_R_ = 7.93 min. ^1^H NMR (400 MHz, CDCl_3_, major rotamer): δ 7.28–6.79 (m, 25H, Ar), 5.11 (d, 2H, *J* = 2.9, OCH_2_), 4.32 (q, 1H, *J* = 6.6, 1’’-H), 3.62 (dd, 1H, *J* = 9.5, 5.7, α-Phe), 3.55 (d, 2H, *J* = 13.9, NCH_2_), 3.47 (d, 2H, *J* = 13.8, NCH_2_), 3.31–3.10 (m, 3H, 1’-H, β-Phe), 3.07 (m, 1H, 2’-H), 2.90 (dd, 1H, *J* = 15.4, 8.4, 1’-H), 2.65 (m, 2H, 3’-H), 1.54 (d, 3H, *J* = 6.5, 2’’-H). ^13^C NMR (75 MHz, CDCl_3_): δ 170.2 (COO), 169.5 (CON), 139.1, 137.9, 136.6, 129.9, 129.8, 129.4, 128.9, 128.8, 128.75, 128.7, 128.5, 128.45, 128.4, 127.3, 126.8, 126.5 (Ar), 67.4 (OCH_2_), 64.0 (α-Phe), 59.1 (C2’), 53.8 (NCH_2_), 52.1 (C1’’), 50.3 (C1’), 36.3 (β-Phe), 34.8 (C3’) 21.5 (C2’’). MS (ES)^+^: 659.35 [M+H]^+^.

##### *N*-(2″*S*-Chloropropanoyl)-N-[2′*R*-dibenzylamino)-3′-phenyl)prop-1′-yl]-L-Phe-OBn (**10**)

Syrup. Yield: 20% (from **8**). Eluents: EtOAc:Hex (1:7). Mixture of rotamers M/m = 2:1. HPLC: t_R_ = 7.88 min (gradient of 5% to 100% of A, in 20 min). ^1^H NMR (400 MHz, CDCl_3_, Major rotamer): δ 7.27–6.91 (m, 25H, Ar), 5.10 (d, 1H, *J* = 12.2 Hz, OCH_2_), 5.03 (d, 1H, *J* = 12.2 Hz, OCH_2_), 4.44 (q, 1H, *J* = 7.0 Hz, 1′-H), 3.55 (d, 2H, *J* = 14.0 Hz, NCH_2_), 3.51 (d, 2H, *J* = 13.9 Hz, NCH_2_), 3.48 (m, 1H, α-Phe), 3.34 (dd, 1H, *J* = 14.1, 5.9 Hz, β-Phe), 3.08 (m, 1H, 2′-H), 3.03 (m, 1H, β-Phe), 2.92 (m, 1H, 1′-H), 2.89 (dd, 1H, *J* = 14.0, 8.6 Hz, 1′-H), 2.76 (dd, 1H, *J* = 13.4, 7.5 Hz, 3′-H), 2.67 (dd, 1H, *J* = 13.4, 6.6 Hz, 3′-H), 1.72 (d, 3H, *J* = 6.5 Hz, 2″-H). ^13^C NMR (75 MHz, CDCl_3_): δ 169.6 (COO), 168.3 (CON), 139.0, 138.2, 135.8, 130.1, 129.6, 128.7, 128.6, 128.55, 128.5, 128.4, 127.3, 126.8, 126.5 (Ar), 67.2 (OCH_2_), 63.2 (α-Phe), 59.2 (C2′), 53.6 (NCH_2_), 50.9 (C1″), 48.9 (C1′), 34.6 (C3′) 34.5 (β-Phe), 21.7 (C2″). MS (ES)^+^: 659.56 [M+H]^+^.

##### *N*-(2″*R*-Chloropropanoyl)-N-[2′*S*-dibenzylamino)-3′-phenyl)prop-1′-yl]-L-Phe-OBn (**11**)

Syrup. Yield: 52% (from **7**). Eluent: EtOAc:Hex (1:6). Mixture of rotamers, M/m = 7:1. HPLC: t_R_ = 7.25 min. ^1^H NMR (400 MHz, CDCl_3_, major rotamer): δ 7.26–6.76 (m, 25H, Ar), 4.93 (s, 2H, OCH_2_), 4.58 (q, 1H, *J* = 6.5, 1’’-H), 3.53 (dd, 1H, *J* = 15.9, 4.7, 1’-H), 3.54 (d, 2H, *J* = 13.9, NCH_2_), 3.50 (d, 2H, *J* = 13.9, NCH_2_), 3.16 (dd,1H, *J* = 13.8, 10.1, β-Phe), 3.10 (dd, 1H, *J* = 13.8, 4.7, β-Phe), 3.00 (m, 1H, 2’-H), 2.89 (dd, 1H, *J* = 10.1, 4.7, α-Phe), 2.68 (dd, 1H, *J* = 13.2/6.6, 3’-H), 2.50 (dd, 1H, *J* = 13.1/8.4, 3’-H), 2.04 (dd, 1H, *J* = 15.9, 5.7, 1’-H), 1.34 (d, 3H, *J* = 6.4, 2’’-H). ^13^C NMR (75 MHz, CDCl_3_): δ 169.3 (COO), 169.2 (CON), 139.1, 138.9, 137.8, 136.0, 129.9, 129.8, 128.8, 128.7, 128.6, 128.5, 128.4, 128.3, 127.4, 126.7, 126.6 (Ar), 67.1 (OCH_2_), 64.8 (α-Phe), 62.4 (C2’), 53.9 (NCH_2_), 52.5 (C1’’), 49.6 (C1’), 34.5 (β-Phe), 34.4 (C3’), 20.5 (C2’’). MS (ES)^+^: 659.35 [M+H]^+^.

##### *N*-(2″*R*-Chloropropanoyl)-N-[2′*R*-dibenzylamino)-3′-phenyl)prop-1′-yl] -L-Phe-OBn (**12**)

Syrup. Yield: 43% (from **8**). Eluents: EtOAc:Hex (1:8). Mixture of rotamers M/m = 3:1. HPLC: t_R_ = 7.84 min (gradient of 5% to 100% of A, in 20 min). ^1^H NMR (400 MHz, CDCl_3_, Major rotamer): δ 7.32–6.92 (m, 25H, Ar), 5.21 (d, 1H, *J* = 12.2 Hz, OCH_2_), 4.91 (d, 1H, *J* = 12.2 Hz, OCH_2_), 4.45 (q, 1H, *J* = 7.0 Hz, 1’’-H), 3.80 (dd, 1H, *J* = 10.1, 4.5 Hz, α-Phe), 3.51 (d, 2H, *J* = 14.0 Hz, NCH_2_), 3.35 (m, 2H, β-Phe), 3.34 (d, 2H, *J* = 14.0 Hz, NCH_2_), 3.28 (dd,1H, *J* = 15.2, 7.1 Hz, 1’-H), 3.12 (m, 1H, 2’-H), 2.68 (dd, 1H, *J* = 13.2, 8.3 Hz, 3’-H), 2.54 (dd, 1H, *J* = 13.2, 6.7 Hz, 3’-H), 2.45 (dd,1H, *J* = 15.4, 4.0 Hz, 1’-H), 1.74 (d, 3H, *J* = 7.0 Hz, 2’’-H). ^13^C NMR (75 MHz, CDCl_3_): δ 169.7 (COO), 168.3 (CON), 139.0, 138.2, 135.8, 130.0, 129.6, 128.75, 128.65, 128.6, 128.45, 128.4, 127.3, 126.9, 126.5 (Ar), 67.3 (OCH_2_), 63.2 (α-Phe), 59.24 (C2’), 53.2 (NCH_2_), 50.9 (C1’’), 48.9 (C1’), 34.6 (C3’), 34.5 (β-Phe), 21.6 (C2’’). MS (ES)^+^: 659.56 [M+H]^+^.
